# Internal vacuum-assisted closure device in the swine model of severe liver injury

**DOI:** 10.1186/1749-7922-7-38

**Published:** 2012-12-05

**Authors:** Christopher B Everett, Bruce W Thomas, Michael Moncure

**Affiliations:** 1 Department of Surgery, The University of Kansas School of Medicine-Wichita, Wichita, Kansas, USA; 2 Department of Surgery, The University of Kansas School of Medicine-Wichita, 929 N. Saint Francis Street, Wichita, Kansas 67214, USA; 3 Department of Surgery, Kansas University Medical Center, Kansas City, Kansas, USA

**Keywords:** Liver trauma, Negative-pressure therapy, Swine model, Hemorrhage

## Abstract

**Objectives:**

The authors present a novel approach to nonresectional therapy in major hepatic trauma utilizing intraabdominal perihepatic vacuum assisted closure (VAC) therapy in the porcine model of Grade V liver injury.

**Methods:**

A Grade V injury was created in the right lobe of the liver in a healthy pig. A Pringle maneuver was applied (4.5 minutes total clamp time) and a vacuum assisted closure device was placed over the injured lobe and connected to suction. The device consisted of a perforated plastic bag placed over the liver, followed by a 15 cm by 15cm VAC sponge covered with a nonperforated plastic bag. The abdomen was closed temporarily. Blood loss, cardiopulmonary parameters and bladder pressures were measured over a one-hour period. The device was then removed and the animal was euthanized.

**Results:**

Feasibility of device placement was demonstrated by maintenance of adequate vacuum suction pressures and seal. VAC placement presented no major technical challenges. Successful control of ongoing liver hemorrhage was achieved with the VAC. Total blood loss was 625 ml (20ml/kg). This corresponds to class II hemorrhagic shock in humans and compares favorably to previously reported estimated blood losses with similar grade liver injuries in the swine model. No post-injury cardiopulmonary compromise or elevated abdominal compartment pressures were encountered, while hepatic parenchymal perfusion was maintained.

**Conclusion:**

These data demonstrate the feasibility and utility of a perihepatic negative pressure device for the treatment of hemorrhage from severe liver injury in the porcine model.

## Front matter

This is a proof-of-concept investigation using the swine model of grade V exsanguinating liver trauma. The aim of the investigation was to determine if the internal application of a modified vacuum-assisted closure device to the injured liver could control hemorrhage.

## Key points

• High grade, exsanguinating liver injury requires rapid control.

• Application of a negative pressure device to exsanguinating liver injury is a variant of "packing" that may offer several advantages.

• In this proof-of-concept investigation using an animal model of liver trauma, application of a negative pressure device rapidly controlled hemorrhage.

## Introduction

The liver is the most commonly injured intraperitoneal organ [1]. Treatment of liver trauma has evolved significantly over the past thirty years and is now often managed non-operatively [2,3]. Operative management, almost exclusively reserved for Grade IV and V injuries, has included such procedures as selective hepatic artery ligation, [4,5] omental packing, anatomic and non-anatomic hepatic resection and deep liver suturing, some of which remain controversial [1,3-6].

Short of formal resection, adequate debridement of non-viable hepatic parenchyma is generally advocated and performed, since it is thought to be vital in minimizing septic complications [1]. Liver transplantation for trauma has been described [7], but largely abandoned and limited only to a few extraordinary cases, guided more by conjecture and circumstance than by evidence.

Perihepatic packing has been utilized to treat liver injury since the Second World War. Success in civilian trauma has revitalized this modality as a temporizing measure to control hemorrhage, particularly in cases complicated by the deadly triad of hypothermia, hypocoagulability and acidosis [8-11]. Similarly, “mesh-wrapping” of the liver to control bleeding, owing to its compressive effect, has been used with some success [12].

Adjuncts to this approach including angiography with selective vessel embolization, computed tomography directed drainage of abscess or biloma, and endoscopic retrograde cholangiopancreatography with biliary stenting have recently been integrated into the nonoperative management strategy of liver trauma with encouraging results [13].

Liver packing, although a life-saving maneuver is not without complications. Placing sponges between the liver and diaphragm to tamponade bleeding compromises venous return, impairing cardiopulmonary function in patients with already limited reserve. Re-bleeding and intraabdominal abscess formation after pack removal has also been described. In patients who require massive resuscitation, visceral edema and elevated intraabdominal pressures may lead to subsequent abdominal compartment syndrome with the use of perihepatic packing.

Abdominal compartment syndrome may cause compromise of cardiac performance and respiratory function, renal function, splanchnic perfusion, and may impair cerebral perfusion [14-17]. The concepts of damage control laparotomy, multiorgan failure, and abdominal compartment syndrome have lead to the use of temporary abdominal closures to allow rapid means of abdominal domain control, in anticipation of delayed, definitive intraabdominal injury repair [13,18,19].

Vacuum assisted closure (VAC), also referred to as negative pressure wound therapy, has gained wide acceptance for use in the management of a range of acute and chronic wounds as well as for temporary abdominal closures in cases of abdominal compartment syndrome and damage control laparotomy [20,21]. VAC therapy combines several features conducive to wound healing including apposition, drainage and coverage. VAC has been successfully utilized to treat numerous and varied conditions including decubitus ulcers, skin grafts, enterocutaneous fistulae, animal and insect bites, osteomyelitis, urologic and perineal wounds, burns, and post-sternotomy sternal wound infections [22-30]. Temporary abdominal closure after damage control laparotomy for abdominal compartment syndrome has been successfully managed using VAC and this modality is now used routinely in our Level I trauma center for such cases.

The porcine or swine model has been used extensively to simulate, experimentally, human liver injury [31-38]. A reproducible Grade V liver injury has been consistently attained in a number of swine model liver trauma studies by the standardized use of a device well described in the trauma and military literature [31,33,34,36-38].

Given the complications associated with traditional hepatic packing, the authors present a novel approach to nonresectional therapy in major hepatic trauma utilizing intraabdominal perihepatic vacuum assisted closure or Liver VAC (L-VAC) therapy in the porcine model. We propose this as a new method for control of hepatic hemorrhage without concomitant cardiopulmonary compromise or development of abdominal compartment syndrome.

## Materials and methods

All experimental methods were conducted in accordance with standard and humane animal laboratory regulations. The study protocol was approved by the Institutional Animal Care and Use Committee at the Kansas University Medical Center.

A healthy, female, 32kg Chester White pig was fasted overnight. The animal was then sedated with intramuscular Telazol (5mg/kg) and Rompun (2mg/kg). General anesthesia was then maintained by inhalational Isoflurane after the animal was orotracheally intubated. The right femoral artery and vein were cannulated via cutdown technique and connected to a continuous monometer. Monitoring included heart rate, blood pressure, hemoglobin-oxygen saturation urine output, end-tidal carbon dioxide or partial pressure of carbon dioxide, respiratory rate, central venous pressure, blood pressure, core temperature, and bladder pressure. Baseline labs consisting of hemoglobin and hematocrit, arterial blood gases, and arterial lactate were obtained from the arterial line and measured at 30 minute intervals throughout the experiment. Intravenous infusion of Lactated Ringer’s crystalloid was used as needed (6mg/kg, titrated) to maintain hemodynamic stability.

**Figure 1:**
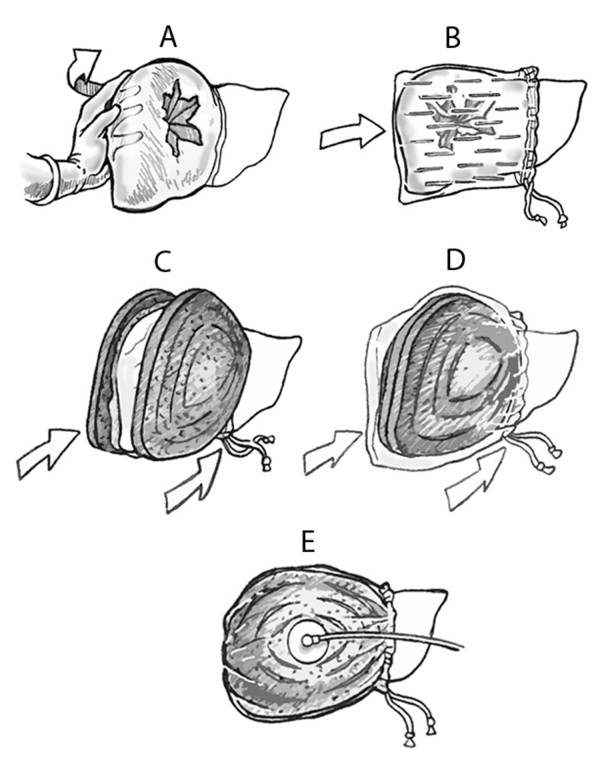
**Illustration demonstrating the procedure for internal application of Liver Vacuum Assisted Closure (L-VAC) device. **(**A**) The injured right lobe is rapidly mobilized. (**B**) A perforated bowel bag is placed over the right lobe. (**C**) A large black sponge is placed over the perforated bag. (**D**) The sponge is covered with a standard bowel bag. (**E**) The Trac pad is applied and connected to suction.

**Figure 2:**
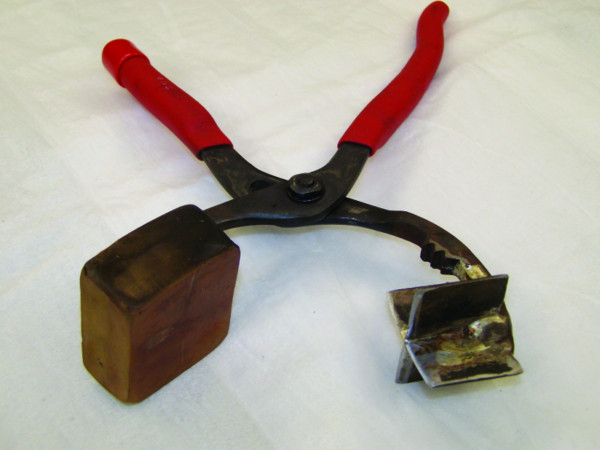
**Photograph of the device used to create the liver injury. **The stellate shape is as described by Holcomb [37].

**Figure 3:**
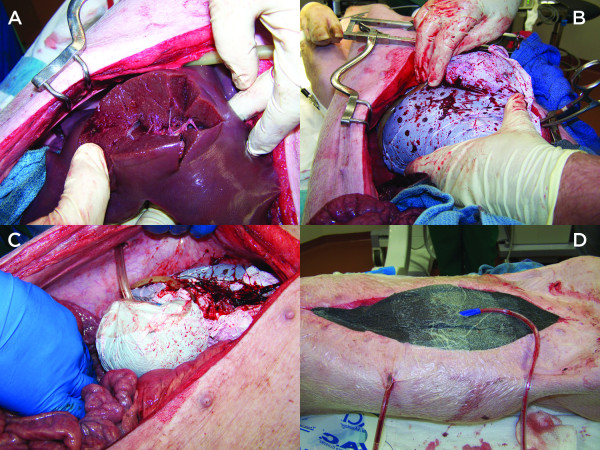
**Intraoperative photographs of liver vacuum assisted closure (L-VAC) device deployment. **(**A**) The liver injury device was applied to the medial lobe of the right liver, moved laterally by 50% and reapplied creating a Grade V injury. (**B**) A perforated bowel bag is placed over the injured lobe from lateral to medial. (**C**) Suction is applied to the device. (**D**) The abdomen was temporarily closed with an abdominal wound VAC device.

A generous midline laparotomy incision was made sharply and entrance to the abdomen was obtained. The bladder was cannulated with a suprapubic catheter and placed to dependent drainage after measurement of bladder pressure. The portal triad structures were mobilized and isolated with a Rumel tourniquet. The right medial lobe of the liver was selected for the site of injury and retracted by manual elevation (Figure 1A). After performing a Pringle maneuver, a standard Grade V liver injury was created according to the method described by Halcomb, Pusateri and Harris [4,31-37]. Briefly, a custom designed clamp with two 4.5-cm sharpened tines configured in the form of an “X” (Figure 2) was positioned over the medial right lobe of the liver on the diaphragmatic surface (Figure 3A). The base plate of the instrument was positioned on the visceral surface. The injury was created by clamping the instrument through the liver parenchyma. The instrument was opened, repositioned medially by 50% and reapplied. The parenchyma was inspected with brief release of the Pringle to verify the severity of the injury (Figure 3A). A perforated plastic bag was placed over the right lobe of the liver (Figure 1B, 3B). A 15 by 15 cm black vacuum sponge was placed over the perforated bag (Figure 1C), followed by a nonperforated bag (Figure 1D). The device was secured medially to the liver using a Rumel tourniquet. The suction pad was applied over a window cut into the nonperforated bag and 150 cm of water suction (110 mmHg) was applied to the device (Figure 1E, 3C). After the device was inspected and found to be without leaks, the Pringle maneuver was released (total clamp time of 4.5 minutes). There was then a reduction to 70 cm of water suction (51 mmHg). Initial and final output from the negative pressure device was measured.

The abdomen was temporarily closed with a second negative pressure device. The intraabdominal contents were covered with a large 10cm ×10cm plastic drape. A large black abdominal sponge was placed over the drape, followed by the suction pad. This negative pressure device was connected to 70cm of water suction (51 mmHg, Figure 3D). After 60 minutes the abdomen was opened and the device was removed and the animal was then euthanized.

## Results

### Injury

Visual inspection of the liver parenchyma confirmed Grade V liver injury according to the solid organ injury scale with visible disrupted portal and hepatic veins (Figure 3A). Brisk, active bleeding consistent with this grade of injury was encountered with brief release of the Pringle maneuver.

### Blood loss

Initial blood loss prior to L-VAC placement was 280 ml (8.75 ml/kg). At initial device placement there was 75ml of immediate blood return. Continued losses after applying the device to suction were negligible over the next 60 minutes. Immediate blood loss after removal of the device was 270 ml (8.4 ml/kg) for a total blood loss of 625ml (19.5 ml/kg) for the entire procedure. Hemoglobin counts were 12.2 g/dl, 11.5g/dl, and 9.6g/dl at 0, 30, and 60 minutes, respectively. No blood products were administered.

### Hemodynamics

**Figure 4:**
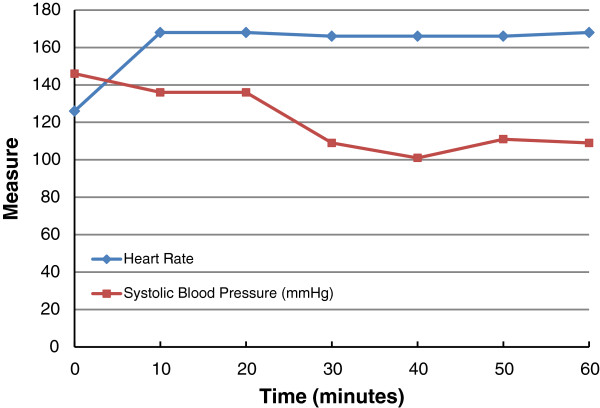
Graph of pulse rate and systolic blood pressure (SBP) as a function of time.

Figure 4 illustrates hemodynamic values during the procedure. The animal remained tachycardic and normotensive throughout the experiment. No cardiovascular compromise was encountered.

### Presence of acidosis

Initial and serial arterial lactate levels were 1.1, 5.8, and 6.8mol/l at 0, 30, and 60 minutes, respectively.

### Intraabdominal pressures

The bladder pressure was 12, 17, and 12 cm H_2_O at 0, 30, and 60 minutes, respectively. Urine output was 73 ml (2.2ml/kg) at 60 minutes.

### Hepatic ischemia

**Figure 5:**
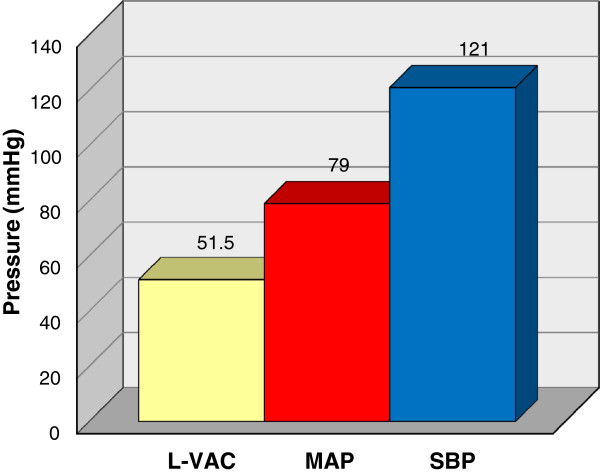
**Average perihepatic vacuum assisted closure pressure (L-VAC), mean arterial pressure (MAP), and systolic blood pressure (SBP). **Hepatic perfusion was maintained by keeping the VAC pressures well below mean arterial pressure and systolic blood pressure.

Mean arterial pressure (MAP) and systolic blood pressure (SBP) averaged 79 mmHg and 121 mmHg, respectively, during L-VAC compression (using 51 mmHg suction) over one hour. Mean hepatic perfusion pressure (MAP minus L-VAC compression pressure) averaged approximately 28 mmHg and the mean systolic perfusion pressure (SBP minus L-VAC compression pressure) averaged approximately 70 mmHg (Figure 5). While this is an indirect surrogate measure for hepatic perfusion pressure, we are confident that it represents a reliable method to confirm adequate hepatic perfusion.

## Discussion

Continued advancements in the management of complex liver injuries have led to an improvement in patient mortality rates. The employment of a multidisciplinary approach encompassing operative and non-operative therapeutic modalities has been crucial to this success. Methods such as packing, hepatic angiography and embolization, and open resection have fallen under scrutiny as investigators seek to overcome the formidable challenge of controlling blood losses in patients in extremis while preventing abdominal compartment syndrome and cardiopulmonary compromise. This study proposes an additional therapeutic technique to the surgeon’s armamentarium by demonstrating the effectiveness of a perihepatic negative pressure device in controlling hemorrhage from severe liver injury in the porcine model.

The feasibility of device placement was demonstrated by maintenance of adequate vacuum suction pressures. Initial seal was obtained at 150 cm of water suction (110 mmHg) and maintained at 70 cm water (51 mmHg) without evidence of vacuum leak. The device was easily deployed with readily available materials, a strength of current therapeutic modalities including perihepatic packing with laparotomy sponges.

Application of this device in clinical practice may be affected by minor anatomic differences between the swine and humans. Specifically, mobilization of the phrenohepatic and triangular ligaments may be necessary to allow for adequate sealing of the device. The author’s personal experience in human cadavers has shown favorable results with no technical difficulties. Given the initial learning curve with this novel application of the L-VAC device, it is the author’s recommendation that clinicians practice in a cadaveric model prior to attempting operative placement in the acute traumatic setting. Careful patient selection is also warranted based on injury location. Injuries to the more medial portions of the liver may impair sealing of the device.

The device demonstrated successful control of ongoing hemorrhage. Significant bleeding was encountered after creation of the injury and prior to control of the porta hepatis, as well as after final removal of the device. Ongoing blood losses after application of the L-VAC device to suction were 75ml, initially, after which they became negligible over the remainder of the 60 minute experiment. Total blood loss was 625 ml (20ml/kg). This corresponds to class II hemorrhagic shock in humans. While no controls were performed comparing this novel method to traditional therapies, the amount of blood loss with the L-VAC compares favorably to that reported in the current literature. Reported mean estimated blood losses approach 3,700ml in swine with similar injures treated with packing and hemostatic bandages.

Hepatic parenchymal perfusion was maintained by keeping L-VAC pressures well below the mean and systolic blood pressure throughout the experiment (Figure 5). The liver appeared well perfused by gross inspection upon removal of the device. The L-VAC provides a theoretical advantage over perihepatic packing by the ability to regulate the amount of pressure applied to the hepatic parenchyma in real-time. To prevent hepatic ischemia, the vacuum setting can be adjusted to the lowest possible setting that allows for sealing and hemostasis. This may be accomplished in the clinical setting by following L-VAC suction canister output and titrating vacuum pressures accordingly.

No post-injury cardiopulmonary compromise was encountered during use of the L-VAC. Venous return to the heart was unaffected as central venous pressures and SBP remained within normal limits throughout the experiment and serum lactate levels elevated in proportion to the level of hemorrhage.

Traditional packing methods rely on compressing the liver between the abdominal wall and the spine for hemostasis. Pressure is also directed posteriorly toward the retroperitoneum and the retrohepatic vena cava. This impediment of venous return is poorly tolerated in the hypovolemic patient and may exacerbate already compromised cardiac output. Given the circular geometry of the L-VAC device, the force vectors are directed inward toward the liver parenchyma. This allows for pressure application to the injured organ without a concomitant decrease in venous return, a distinct advantage over traditional perihepatic packing.

Perihepatic packing has also been shown to result in pathologic intra-abdominal hypertension [39]. In this study, abdominal compartment pressures remained low throughout the procedure. Urine output was commensurate with the level of hypovolemia, and end tidal CO2 levels remained constant. In addition, the bowel and bladder appeared well perfused upon device removal. With intraabdominal packing, temporary abdominal closure does not necessarily prevent the development of abdominal compartment syndrome. Prior investigators have demonstrated that unpacking the abdomen results in a significant improvement in cardiopulmonary function as well as renal and intestinal blood flow [39]. Monitoring of bladder pressures, urine output, and end tidal carbon dioxide levels with subsequent titration of vacuum pressures on the abdominal device is recommended.

The applicability of the swine model for human liver injury has been well described in the literature. This model, however, is not without its limitations. The compression of the portal inflow during creation of the liver laceration minimized initial blood losses. In the clinical setting, uncompensated hypovolemic shock may result in the ‘bloody vicious cycle’ of hypothermia, acidosis, and coagulopathy. Obtaining hemostasis from bleeding viscera in the face of these physiologic derangements can be quite challenging. In this regard, the model used for this experiment was artificial given that the pig was well compensated hemodynamically, with functioning coagulation cascades. However, given the mechanism of action of the VAC device, the authors contend that L-VAC placement may be the ideal therapy for control of hemorrhage in such cases. Consideration is being given to repeating this experiment in animals that are hypothermic and coagulopathic.

Future areas of investigation should be directed toward comparing this innovative method to well-established therapies such as packing, mesh wrapping, and application of hemostatic agents.

In summary, these data demonstrate the feasibility and utility of a perihepatic negative pressure device for the treatment of hemorrhage from severe liver injury in the porcine model. This method is potentially applicable in the clinical setting and may afford advantages over traditional damage control procedures such as perihepatic packing.

## Competing interests

The authors declare that they have no competing interests.

## Authors’ contributions

BT initially conceived the study idea. BT, CE, and MM were all involved in the study design and procedure. CE drafted the initial case report. Case report revisions and final report submission were all conducted by BT, CE, MM. All authors read and approved the final manuscript.

## Financial disclosure

This study was funded in part by funds from the Kansas University Medical Center, and the Wesley Medical Center Trauma Research Fund.

## Institutional animal use and care committee approval

This study was approved for implementatin by the IACUC of the Kansas University Medical center.
